# Supplementary material for: Long-term health-related quality of life, healthcare utilisation and back-to-work activities in intensive care unit survivors: Prospective confirmatory study from the Frisian aftercare cohort
Source: PLoS One. 2022 Sep 7;17(9):e0273348. doi: 10.1371/journal.pone.0273348 (PMC9451092; doi:10.1371/journal.pone.0273348)
Supplement: S1 Table — (DOCX) [file pone.0273348.s001.docx]

**S1 table. Characteristics of lost-to-follow-up**

| **Characteristics** | **Included in analysis, n=65** | **Lost-to-follow-up at 12 m, n=16** | **p-value** |
| --- | --- | --- | --- |
| **Demographic factors** |  |  |  |
| Female, n (%) | 16 (25%) | 10 (63%) | **0.006** |
| Age | 66 [57-74] | 72 [68-78] | 0.124 |
| BMI (kg/m2) | 27 [24-31] | 26 [24-32] | 1.000 |
| APACHE III | 76 [58-97] | 82 [49-115] | 0.859 |
| CFS (1-9) | 3 [2-4] | 3 [3-5] | 0.321 |
| **Comorbidities** |  |  |  |
| Malignancy, n (%) | 6 (9%) | 4 (25%) | 0.102 |
| Diabetes, n (%) | 11 (17%) | 2 (13%) | 1.000 |
| COPD, n (%) | 8 (12%) | 2 (13%) | 1.000 |
| CVA, n (%) | 6 (9%) | 0 (0%) | 0.593 |
| CKD, n (%) | 6 (9%) | 5 (32%) | **0.036** |
| Multicomorbidity, n (%) | 7 (11%) | 4 (25%) | 0.214 |
| Psychiatric history, n (%) | 15 (23%) | 5 (31%) | 0.526 |
| **Aetiology** |  |  |  |
| Admission, n (%) |  |  |  |
| Medical | 34 (52%) | 11 (69%) | 0.454 |
| Elective surgical | 16 (25%) | 2 (13%) |  |
| Acute surgical | 15 (23%) | 3 (19%) |  |
| Sepsis, n (%) | 14 (22%) | 4 (25%) | 0.746 |
| CPR, n (%) | 10 (15%) | 2 (13%) | 1.000 |
| Delirium, n (%) | 24 (37%) | 3 (19%) | 0.239 |
| **ICU morbidity** |  |  |  |
| LOS ICU | 5 [4-10] | 5 [3-20] | 0.765 |
| Mechanical ventilation (days) | 3 [1-6] | 2 [0-12] | 0.641 |
| Renal replacement therapy (CVVH) | 11 (17%) | 2 (13%) | 1.000 |
| **Post-ICU** |  |  |  |
| ICU readmission within 1 year, n (%) | 2 (3%) | 0 (0%) | 1.000 |
| Rehabilitation intensity |  |  |  |
| No rehabilitation | 12 (19%) | 11 (69%) | **0.001** |
| Self-initiated or primary care | 17 (26%) | 2 (13%) |  |
| Cardiac rehabilitation program | 20 (31%) | 0 (0%) |  |
| General rehabiliation center | 14 (22%) | 3 (19%) |  |
| Nursing home | 2 (3%) | 0 (0%) |  |
