# Supplementary material for: Long-term health-related quality of life, healthcare utilisation and back-to-work activities in intensive care unit survivors: Prospective confirmatory study from the Frisian aftercare cohort
Source: PLoS One. 2022 Sep 7;17(9):e0273348. doi: 10.1371/journal.pone.0273348 (PMC9451092; doi:10.1371/journal.pone.0273348)
Supplement: S2 Table — (DOCX) [file pone.0273348.s002.docx]

**S2 table. HRQoL domain scores at baseline and at 3, 6, and 12m**

| **RAND-36 domain scores (0-100)** | **All, n=65** | **NR at 12 months, n=36 (55%)** | **R at 12 months,  n=29 (45%)** | **p-value** |
| --- | --- | --- | --- | --- |
| **Baseline** |  |  |  |  |
| Physical functioning | 65 [45-95] | 55 [29-81] | 95 [65-100] | **<0.001** |
| Social functioning | 88 [63-100] | 88 [50-100] | 88 [63-100] | 0.132 |
| Role functioning (physical   problems) | 50 [0-100] | 25 [0-81] | 100 [25-100] | **0.011** |
| Role functioning (emotional   problems) | 100 [67-100] | 100 [67-100] | 100 [83-100] | 0.602 |
| Mental health | 88 [68-96] | 82 [67-93] | 88 [72-96] | 0.204 |
| Energy/Fatigue | 70 [40-80] | 58 [35-75] | 70 [60-85] | **0.017** |
| Bodily pain | 78 [55-100] | 71 [43-100] | 88 [68-100] | **0.049** |
| General health perception | 60 [44-85] | 50 [30-75] | 70 [50-90] | **0.003** |
| Health change | 50 [25-50] | 25 [19-50] | 50 [25-50] | 0.19 |
| **3m after discharge** |  |  |  |  |
| Physical functioning | 60 [35-90] | 40 [20-58] | 88 [79-95] | **<0.001** |
| Social functioning | 76 [50-96] | 63 [44-86] | 92 [75-100] | **0.003** |
| Role functioning (physical   problems) | 25 [0-75] | 0 [0-69] | 50 [25-100] | **0.007** |
| Role functioning (emotional  problems) | 100 [67-100] | 100 [67-100] | 100 [84-100] | 0.103 |
| Mental health | 88 [60-97] | 70 [54-95] | 88 [72-100] | 0.165 |
| Energy/Fatigue | 60 [35-80] | 55 [33-70] | 75 [49-81] | **0.030** |
| Bodily pain | 78 [45-100] | 68 [38-90] | 80 [58-100] | 0.154 |
| General health perception | 60 [50-75] | 50 [40-63] | 70 [59-100] | **<0.001** |
| Health change | 50 [25-75] | 50 [25-75] | 50 [38-75] | 0.257 |
| **6m after discharge** |  |  |  |  |
| Physical functioning | 65 [35-90] | 43 [25-61] | 90 [69-95] | **<0.001** |
| Social functioning | 80 [66-100] | 75 [62-95] | 95 [79-100] | **0.011** |
| Role functioning (physical   problems) | 50 [0-88] | 25 [0-50] | 75 [25-100] | **0.003** |
| Role functioning (emotional   problems) | 100 [67-100] | 100 [67-100] | 100 [100-100] | 0.207 |
| Mental health | 80 [62-96] | 80 [60-92] | 80 [63-97] | 0.5 |
| Energy/Fatigue | 63 [45-80] | 58 [40-75] | 75 [54-85] | 0.069 |
| Bodily pain | 84 [60-100] | 78 [45-100] | 100 [78-100] | **0.014** |
| General health perception | 60 [42-75] | 55 [40-70] | 70 [55-80] | **0.006** |
| Health change | 50 [25-50] | 50 [25-50] | 50 [25-75] | 0.235 |
| **12m after discharge** |  |  |  |  |
| Physical functioning | 65 [30-90] | 35 [15-55] | 95 [83-95] | Na^a^ |
| Social functioning | 88 [63-100] | 75 [63-88] | 96 [74-100] | **0.004** |
| Role functioning (physical   problems) | 50 [0-100] | 0 [0-50] | 100 [75-100] | **<0.001** |
| Role functioning (emotional   problems) | 100 [42-100] | 100 [33-100] | 100 [67-100] | 0.146 |
| Mental health | 88 [63-100] | 80 [53-92] | 96 [80-100] | **0.006** |
| Energy/Fatigue | 60 [48-85] | 50 [40-69] | 80 [60-90] | **<0.001** |
| Bodily pain | 88 [68-100] | 73 [48-100] | 100 [78-100] | **0.001** |
| General health perception | 60 [35-84] | 55 [35-65] | 75 [50-90] | **0.001** |
| Health change | 63 [50-100] | 50 [25-100] | 75 [50-100] | 0.236 |

**^a^used as allocation value**
